# Outcomes in Cardiac Surgery Based on Preoperative, Mean Intraoperative and Stratified Cerebral Oximetry Values

**DOI:** 10.7759/cureus.17123

**Published:** 2021-08-12

**Authors:** Sean R Bennett, Abdulkarim W Abukhodair, Mohammed S Alqarni, Jose A Fernandez, Andres J Fernandez, Miriam R Bennett

**Affiliations:** 1 Anaesthesiology, Cardiac and Intensive Care, King Saud Bin Abdulaziz University for Health Sciences (KSAU-HS), Jeddah, SAU; 2 Medical Intern, King Saud Bin Abdulaziz University for Health Sciences (KSAU-HS), Jeddah, SAU; 3 Anesthesia and Critical Care, King Saud Bin Abdulaziz University for Health Sciences (KSAU-HS), Jeddah, SAU; 4 Medicine, Luis Razzetti School of Medicine, Central University of Venezuela, Caracas, VEN; 5 Respiratory Medicine, Manchester University NHS Foundation Trust, Manchester, GBR

**Keywords:** cerebral ischaemia, cerebral oximetry, cardiac surgery, renal dysfunction, length-of-stay

## Abstract

Introduction: Cardiac surgery is associated with significant morbidity and longer length-of-stay (LOS) than most other surgeries. Regional cerebral oximetry (rSO_2_) using near-infrared spectroscopy (NIRS) on the patient’s forehead monitors cerebral oxygenation during surgery and cardiopulmonary bypass (CPB). Its purpose is to detect and manage periods of cerebral hypoxia which may otherwise go undetected, thereby reducing morbidity. But outcomes have been inconsistent, and not all cardiac departments have adopted this non-invasive, simple-to-use technology. We aimed to study the efficacy of our use of rSO_2_ by recording seven outcomes for each patient according to their preoperative rSO_2_, the mean intraoperative rSO_2_, and four ischemic thresholds during surgery.

Method: This is a retrospective audit of cardiac surgical patients in whom a protocol was used to maintain rSO_2_ above the preoperative value and studied seven major morbidity outcomes. Cerebral oximetry data were recorded for each patient and analyzed for six variables: preoperative baseline rSO_2_, mean intraoperative rSO_2,_ and four ischemic thresholds defined as an area under the curve (AUC) in minutes% below the baseline rSO_2_,minus 10% below the baseline, minus 20% the below baselineand minus 50% below baseline. Outcomes examined were: delirium, stroke, postoperative rise in creatinine of 50 mmol, absolute creatinine of 200 mmol, need for new renal replacement therapy (RRT), hospital LOS and inpatient mortality.

Results: Complete data were available for 166 patients. Lower mean preoperative rSO_2_ was associated with stroke (p=0.031), mild and severe renal dysfunction (p=0.045 and p=0.036), death-in-hospital (p=0.027) and prolonged hospital LOS (p=0.005). Lower mean intraoperative rSO_2_ during surgery was associated with the outcomes of renal dysfunction, mild (p=0.027), moderate (p=0.003) or severe (p=0.002), death-in-hospital (p=0.003) and prolonged hospital LOS (p=0.015). Of the four ischemic thresholds defined, only new RRT occurring at minus 20% and minus 50% below baseline was significant.

Conclusion: Lower preoperative rSO_2_ and mean intraoperative rSO_2_ were associated with poor outcomes, notably leading to a significant increase in hospital LOS. Mild degrees of cerebral ischemia below the baseline and minus 10% of the baseline during surgery were well tolerated.

## Introduction

Although cardiac surgery has become safer, morbidity and mortality remain significant in both coronary and valvular surgery [1,2]. Patients can be swayed towards less invasive options offered by interventional cardiology even if the end product is inferior. When cerebral oximetry became widely available in 2007, there was hope that cerebral oximetry would significantly improve the safety of cardiopulmonary bypass (CPB), which differentiates cardiac surgery from other major surgeries [3]. Cerebral oximetry is a real-time technique to non-invasively measure cerebral oxygenation using near-infrared spectroscopy (NIRS). In adults, probes are adhered to the forehead and transmit near-infrared light into the frontal cerebral cortex. The reflected signal gives a measure of the regional cerebral oximetry (rSO2) of the hemoglobin in that area of the brain. It is similar to peripheral pulse oximetry but uses reflectance rather than absorption technology [4]. The initial focus for improvement was on two principal areas - neurological injury and major organ morbidity for which the brain would act as an index organ [3]. Both neurological injury and major organ morbidity are more common after cardiac surgery than non-cardiac surgery [5]. Despite high hopes, during the past 15 years, there has been inconsistency in demonstrating significant improvement in any of these outcomes with NIRS monitoring [6]. There are many reasons for this; major events are uncommon, the main predictor of postoperative morbidity is preoperative morbidity and only a small area of the frontal cortex is scanned by the NIRS. However, some studies have shown better outcomes using NIRS [3,7]. One of the more consistent findings using cerebral oximetry is a reduction in intensive care and hospital LOS [7,8]. During CPB, there is no other non-invasive way to directly monitor cerebral oxygenation or any other major organ oxygenation. Because the NIRS value for rSO2 on the screen represents oxygen supply and demand, we can use rSO2 as a mixed venous oxygen saturation for the brain. Also, uncertainty remains as to what degree of ischemic exposure is harmful and what level of cerebral desaturation should be a trigger to intervene. What rSO2 is a ‘safe value' [9]? Our study aimed to determine the efficacy of our use of rSO2 monitoring by recording neurological, renal, and LOS outcomes for each patient and correlating these with the patient’s preoperative rSO2, the mean intraoperative rSO2, and four ischemic thresholds during surgery. This would provide information on whether NIRS was providing prognostic data and/or data on the intraoperative management of oxygenation.

## Materials and methods

This retrospective audit included patients requiring CPB between 2017 and 2020 at the King Faisal Cardiac Centre, Jeddah, Saudi Arabia. Ethical approval was obtained from the King Abdullah International Medical Research Center, reference JED-21-427780-15612. Regional cerebral oximetry (rSO2) values were recorded using a cerebral oximetry monitor (INVOS, 5100, Somanetics/Covidien Corporation, Troy, MI, USA).

Bilateral oximetry probes were placed on the forehead before the induction of anesthesia with the patient breathing room air (baseline rSO2). Regional cerebral oximetry was then recorded as a continuum with values up-dating on the screen until transfer to the Intensive Care Unit (ICU) at which point the probes were removed and rSO2 recording stopped. At the end of each case, the rSO2 data was downloaded and analyzed using software supplied by Covidien.

During the CPB and surgery, for all patients, as standard practice, a protocol was used to keep the rSO2 value at or above baseline using standard interventions shown in Table 1. The techniques used to maintain rSO2 were based on a protocol validated in an earlier study [8].

**Table 1:** Intervention protocol when rSO2 values fall below the preoperative baseline PaCO_2_ - Arterial carbon dioxide tension, SaO_2_ - Peripheral oxygen saturation, FiO_2_ - Fractional inspired oxygen, CPB - Cardiopulmonary bypass

Checklist if rSO_2_ falls below baseline		Intervention
1.	PaCO_2 _<5.3 kPa	Alter ventilation keeping PaCO_2_ at 5.3 kPa
2.	Mean arterial pressure <65 mmHg	Depending on situation vasoconstrictor, volume, or inotrope to keep MAP >65 mmHg
3.	SaO_2_ less than 94%	Increase FiO_2_
4.	Depth of anesthesia	Maybe appropriate to increase depth of anesthesia
5.	Haematocrit	Consider transfusion to hematocrit 27%
6.	Position of aortic cannula and head position.	Usually detected as soon as CPB is started
7.	Re-calculate pump flow on CPB	Check flow (2.4 l/m^2^/min) and venous return
8.	No response to above	Discuss surgical plan with surgeon

The software allowed us to calculate the mean preoperative rSO2, the mean rSO2 for the whole case, and time spent at the four decremental levels of ischemic exposure as in Table 2.

**Table 2:** The six rSO2 variables analyzed for each patient rSO_2_ - Regional cerebral oximetry, AUC - Area under the curve

1.	Preoperative (baseline) rSO_2_	For each patient awake breathing air
2.	Mean intraoperative rSO_2_	Mean rSO_2 _throughout the surgery
3.	Time below preoperative rSO_2_	AUC for any fall below baseline
4.	Threshold rSO_2_ minus 10%	AUC for any fall more than 10% below baseline
5.	Threshold rSO_2_ minus 20%	AUC for any fall more than 20% below baseline
6.	Threshold rSO_2_ minus 50%	AUC for any fall more than 50% below baseline

Once a threshold level was chosen the software calculates the number of minutes and the percentage fall (minutes %) below the chosen threshold. This produces an area under the curve (AUC) value in minutes %.

Outcome data were obtained from the comprehensive electronic medical records system ‘Best Care’, Health Informatics Company, South Korea. Postoperative outcome data comprised of: neurological dysfunction- stroke (confirmed by CT scan) and delirium (documented in medical notes and prescribed haloperidol); renal dysfunction based on Society of Thoracic Surgeons criteria as mild (rise in creatinine of 50 mmol from baseline value), moderate (absolute creatinine value of >200 mmol) and severe [new renal replacement therapy (RRT)]; hospital LOS in days and mortality.

Statistical analysis

Analysis was undertaken using SPSS version 23 and R version 4.0.3 with support from a clinical statistician. Binary outcomes were assessed using Mann-Whiney U and continuous outcomes were assessed using the Student T-test.

Analysis of hospital LOS was done by three different methods as suggested by Harhay et al. [10]. Initial analysis was done using Spearman Rank, first counting LOS up to the day of death and then excluding patients who died in the hospital. Second, we used a Cox analysis and censored those who experienced in-patient mortality. Finally, we undertook a time-to-discharge analysis using a competing risk model, accounting for in-patient mortality as a competing risk.

## Results

Of the 201 CPB patients operated on we had complete records for 166 patients. Demographics are displayed in Table 3.

**Table 3:** Patient Characteristics CABG - Coronary artery bypass graft

	Total number n=166 (%)
Males	121 (73)
Females	45 (27)
Age average years	58.4
Body Mass Index average	29.1
Weight average	76 kg
Ejection Fraction % median	52%
Diabetes	115 (69)
Hypertension	104 (63)
Dyslipidaemia	72 (43)
Renal dysfunction (creatinine>112 mmol)	65 (39)
Type of operation CABG only	118 (71)
Valve only	33 (20)
Valve and CABG	9 (5)
Other	6 (4)

**Figure 1:**
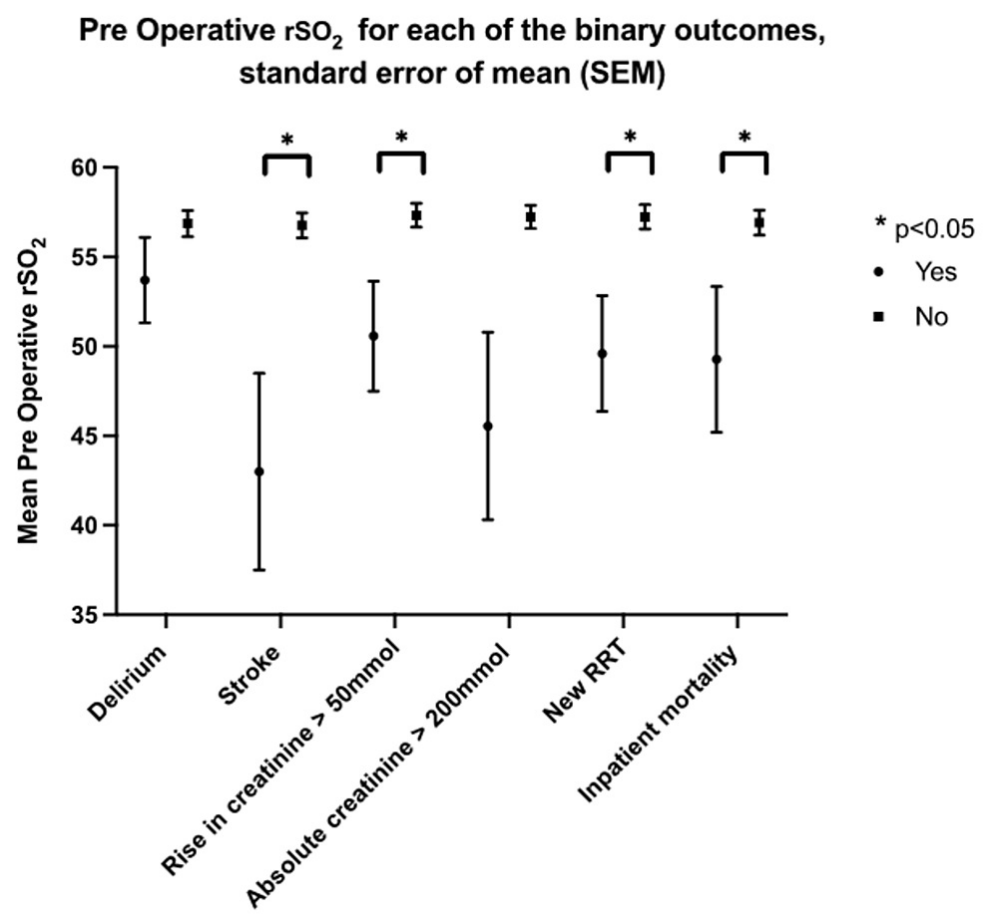
Mean preoperative rSO2 and binary outcomes

Lower preoperative baseline rSO2 was seen in those patients who developed stroke (p=0.031), who suffered mild or severe renal dysfunction (rise in creatinine >50 mmol p=0.045, new RRT p=0.036); and who died in hospital (p=0.027) (Figure 1).

**Figure 2:**
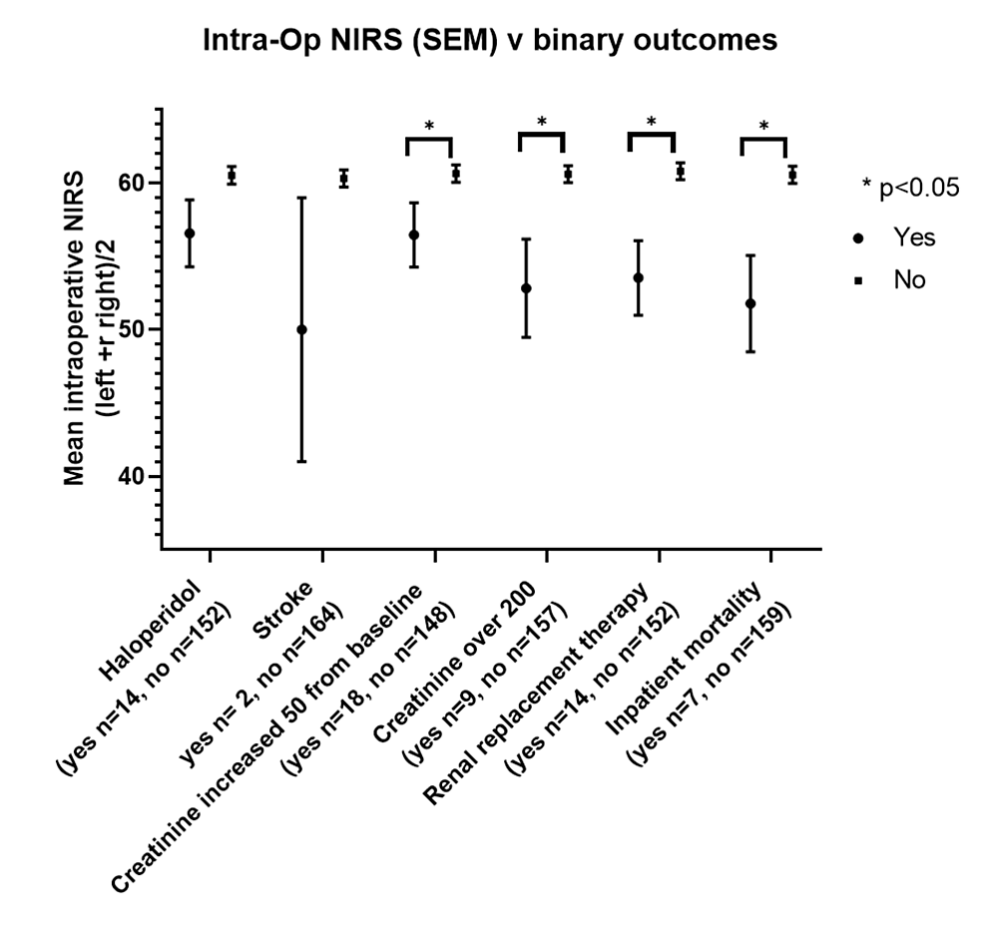
Mean intraoperative rSO2 and binary outcomes

Lower mean intraoperative rSO2 during surgery was seen in those patients who developed: any degree of renal dysfunction (creatinine rise >50 mmol p=0.027, absolute creatinine >200 mmol p=0.003, new RRT p=0.002) and in-patient mortality (p=0.003) (Figure 2).

**Figure 3:**
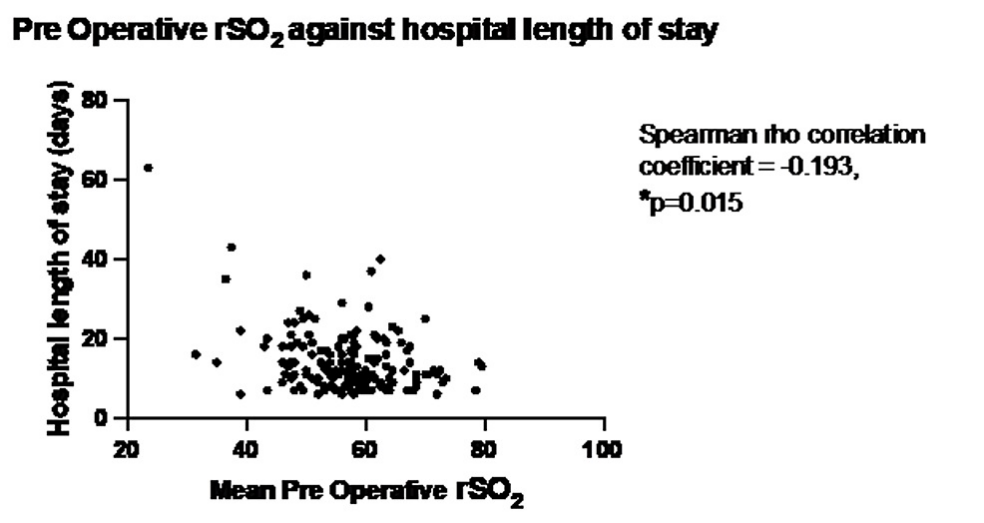
Mean preoperative rSO2 and hospital LOS

Lower preoperative baseline rSO2 was associated with an increased hospital LOS (p=0.005) (Figure 3).

**Figure 4:**
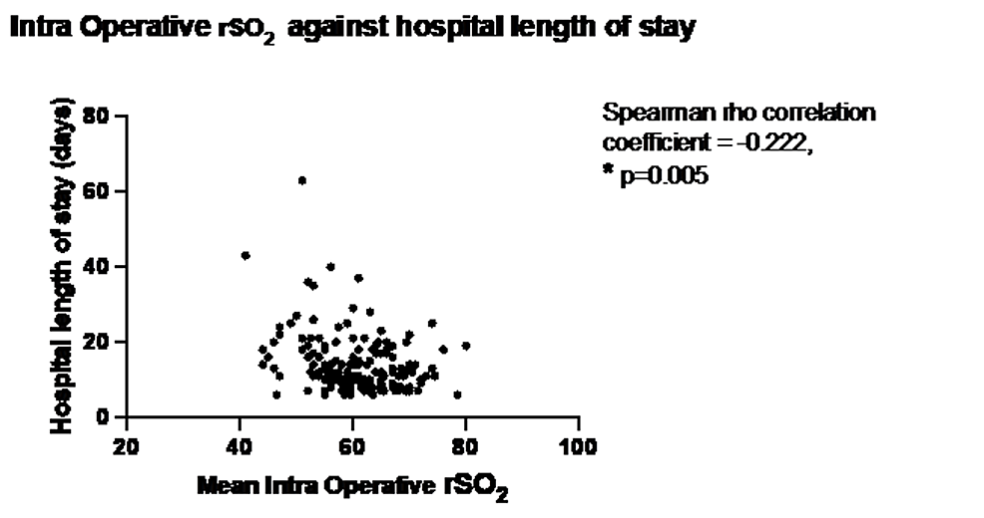
Mean intraoperative rSO2 and hospital LOS

Lower mean intraoperative rSO2 was also associated with an increased hospital LOS (p=0.015) (Figure 4).

In all three different statistical methods used to analyze LOS the preoperative rSO2 and mean intraoperative rSO2 demonstrated a small but statistically significant correlation (Table 4).

**Table 4:** Three statistical models for rSO2 and ischemic thresholds against hospital LOS

	Spearman Rank		Cox	Competing Risk Model
	Including all patients(n=166)	Excluding those with inpatient mortality (n=159)	(n=166)	(n=166)
Preoperative rSO_2_	-0.214 (p=0.006)*	-0.193 (p=0.015)*	1.033 (p<0.01)*	1.034 (p<0.01)*
Intraoperative mean rSO_2_	-0.225 (p=0.004)*	-0.222 (p=0.005)*	1.038 (p<0.01)*	1.039 (p<0.01)*
AUC below baseline rSO_2_	0.119 (p=0.125)	0.122 (p=0.127)	1.000 (p=0.155)	1.000 (p=0.138)
AUC 10% below baseline rSO_2_	0.134 (p=0.084)	0.130 (p=0.104)	1.000 (p=0.137)	1.000 (p=0.121)
AUC 20% below baseline rSO_2_	0.135 (p=0.084)	0.108 (p=0.174)	1.000 (p=0.283)	1.000 (p=0.295)
AUC 50% below baseline rSO_2_	-0.089 (p=0.257)	-0.088 (p=0.268)	1.075 (p=0.501)	1.083 (p=0.461)

**Figure 5:**
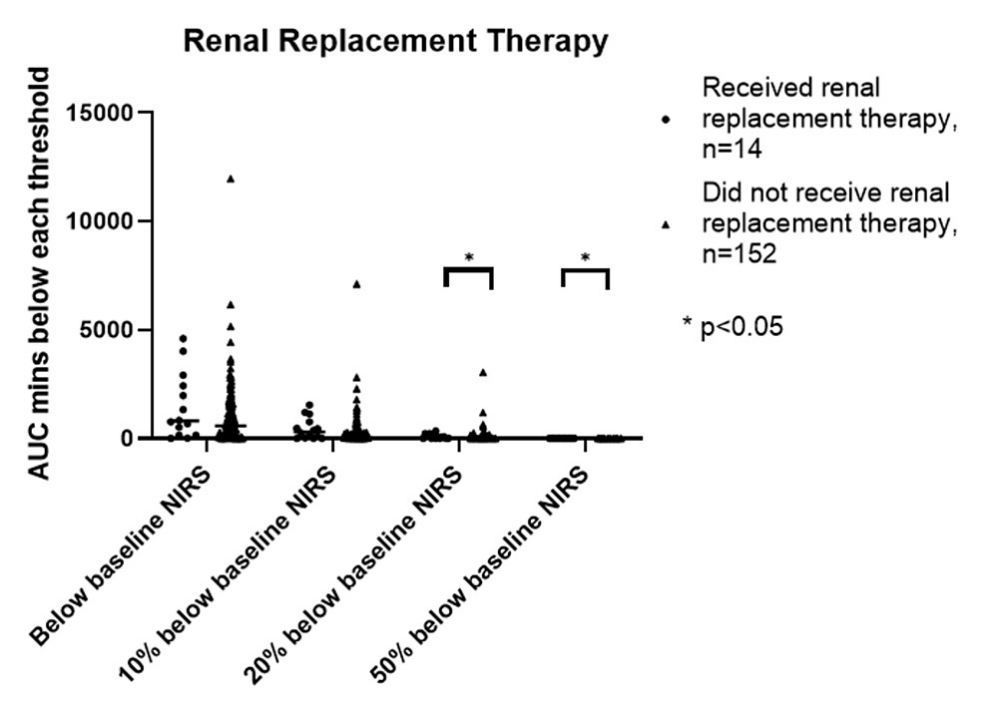
The four ischemic thresholds and the need for renal replacement therapy AUC - Area under the curve. This is the minutes% spent below each threshold.

The time spent under each rSO2 threshold, which can be considered as a marker for ischemic exposure, was statistically significant only for the outcome of RRT at minus 20% and minus 50% below the preoperative (baseline) rSO2 (p=0.006 and p=0.002 respectively), but numbers of patients reaching these thresholds receiving RRT were small (n=7 and n=2 respectively) (Figure 5).

**Figure 6:**
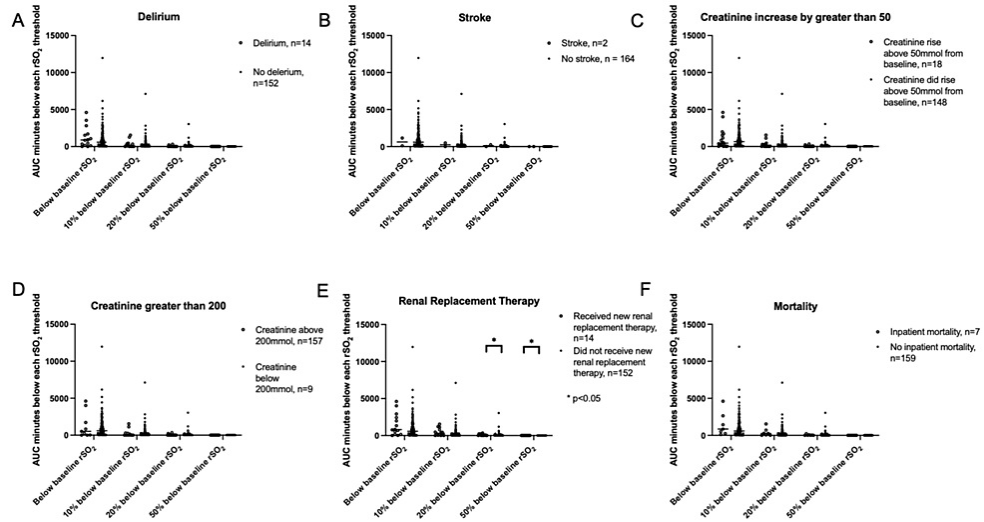
All outcomes against the four ischemic thresholds

There were no statistically significant differences for ischemic exposure below the preoperative rSO2 and minus 10% for any outcome measures (Figure 6).

## Discussion

Our data set showed outcomes in keeping with those seen in the Society Thoracic Surgeons (STS) database of over 500,000 coronary artery bypass graft (CABG) patients with similar mortality at 4.2% (3.1% STS) and stroke at 1.2% (1.6% STS). However, 30-day renal dysfunction was notably better at 0% (3.5% STS) [11]. Although 39% of patients suffered some level of renal dysfunction preoperatively, none required new RRT at 30 days. It is possible that early mortality prevented us from seeing permanent RRT but for our mix of surgical procedures, our mortality was not high. We could therefore speculate that the intraoperative ischemic burden on the kidneys was of severity from which the patients were able to recover.

Mean (SD) preoperative rSO2 for the whole data set was 56.2% (9%) in keeping with that reported by other studies [12]. All of our patients were actively managed under anesthesia to maintain rSO2 values at or above the preoperative baseline, as outlined in Table 1. Thus, we would expect most patients to have an intraoperative mean rSO2 higher than baseline, with the majority of patients not spending significant time below any of the four rSO2 thresholds.

Previous work has suggested that preoperative rSO2 below 50% was a strong predictor of poor postoperative outcomes [12]. This is in keeping with our data which showed that no patients with a preoperative rSO2 > 57% suffered any level of renal impairment.

159 of 166 patients had a mean intraoperative rSO2 of above 60%, none of this group developed renal impairment or stroke and there was only one death. This patient had been discharged to the ward for five days, was ready to go home, when he developed a sternal wound infection and eventually died in multi-organ failure. We believe this aspect of NIRS monitoring during the surgery is an indication of its value, as the judicious application of the interventions shown in Table 1 will ensure better oxygenation throughout the surgery even in patients who start with a low baseline. Although all the interventions shown in Table 1 are often used, the most common corrections that are made to maintain high rSO2 values are to PaCO2, hemoglobin, and arterial blood pressure. Often the changes are not dramatic and would go unnoticed but for the fall in rSO2. One less common correction but sometimes very dramatic is the position of the CPB aortic-cannula which can be poorly positioned causing directional blood flow that compromises the cerebral circulation. This results in a sudden fall in rSO2 while little else changes and is immediately corrected by the re-positioning of the CPB aortic-cannula 

The significance of the higher preoperative rSO2 and a mean rSO2 >60% is also demonstrated in reduced hospital LOS, most likely due to the reduced ischemic burden minimizing organ dysfunction postoperatively. LOS is an outcome measure that has been consistently associated with the use of cerebral oximetry [12]. However, the use of LOS in the ICU setting is not easy to define or analyze given the confounding effect of mortality which can cause misleading results [10]. We accounted for mortality within the LOS analysis using three different statistical models and examined for concordance in the results. All three models showed a statistically significant improvement of higher preoperative and mean rSO2 values leading to shorter LOS. 

Looking at outcomes for patients that spent time below the rSO2 ischemic thresholds, the results indicate that periods of ischemic exposure, confined to below the preoperative baseline and below minus 10%, are well tolerated if the mean for the case is kept above rSO2 of 60%.

At the lower ischemic thresholds, there was a significant difference in RRT for patients who drop below the minus 20% and minus 50% thresholds. But the number of patients reaching these thresholds was small and time spent here was mostly short. This may be due to the rapid clinical response to a decreasing rSO2 as a drop in rSO2 to minus 20% and minus 50% below baseline can be prevented or reversed quickly if the entire team (anesthesia, surgical, and perfusion) is engaged in the management.

Only four patients had a drop in rSO2 of minus 50% from baseline. A drop of 50% from baseline has been considered a level at which organ damage is likely to occur, possibly more important is the total time and degree of ischemia (minute %) of accumulated exposure throughout the surgery. Slater has quoted 3,000 minutes % as a threshold level beyond which ischemic damage is highly likely to be seen [7]. We had 8 patients who accumulated >3,000 minute% below baseline despite our management protocol; two required RRT, one of whom died, which supports this idea. However, it may be that a patient who starts with a high baseline, say rSO2 70%, is able to tolerate a 50% reduction to rSO2 35%, whereas the patient who starts lower, say 36%, will be adversely affected by rSO2 values of 18%. In this regard, a figure of 3,000 minute% is not practical as it depends on the baseline value and is of limited intraoperative use as it is derived retrospectively.

If the cardiac team can consistently achieve rSO2 values above baseline the risk of major organ morbidity and death may be dramatically reduced. We believe that far from being an unmodifiable risk, cerebral oximetry is an important non-invasive monitor to reduce the burden of mortality and postoperative organ dysfunction commonly associated with ischemic exposure during cardiac surgery.

This data supports the fact that baseline rSO2 is itself an important prognostic indicator. Is the preoperative rSO2 itself modifiable? It can be increased by raising the inspired oxygen and patients who fail to improve with pre-oxygenation who have a worse prognosis [12]. In our data set, we did not document the change in rSO2 with oxygenation.

Another discussion is the concept of the brain as an index organ [3]. The theory being that rSO2 acts in a similar way to mixed venous oxygen saturation values [13]. However, to be an index organ it should ideally give a warning at the initiation of any ischemic damage occurring. It may be that cerebral oximetry is able to detect a drop in rSO2 which, thanks to autoregulation, leads to preserved cerebral function at the expense of other organs. This would be supported by our data which showed the most significant differences in intraoperative rSO2 that tended to result in worse renal outcomes, and not in cerebral outcomes such as stroke or delirium.

Strengths and limitations

A limiting factor to the analysis was the cohort size of 166, which resulted in only very small numbers of patients in outcomes groups such as stroke (n=2), limiting the ability to statistically show a difference between predictive variables. Another factor was that we did not collect data on the success of interventions carried out as a result of the protocol to maintain rSO2 above baseline. One strength of this study is the careful consideration of mortality and the effect on the analysis of LOS. Also, the surgical procedure variation in a consecutive, unselected patient group makes the data set a real-life representation of clinical practice. 

## Conclusions

Lower preoperative and intraoperative rSO2 values in adult cardiac surgery can be used to predict poorer renal outcomes and increased hospital LOS. Our protocol keeping rSO2 above baseline was practical and we believe it is the optimal strategy. Also, decrements of rSO2 below the baseline and minus 10% were not associated with poor outcomes, being well tolerated. Although not all outcomes were adversely affected, a drop in rSO2 to minus 20% and minus 50% thresholds were associated with increased RRT. Routine use of rSO2 in adult cardiac surgery is beneficial in preoperative risk stratification, intraoperative management, and in determining the likely course of postoperative recovery.

## References

[1] Shahian DM, O'Brien SM, Filardo G (2009). The Society of Thoracic Surgeons 2008 cardiac surgery risk models: part 1--coronary artery bypass grafting surgery. Ann Thorac Surg.

[2] O'Brien SM, Shahian DM, Filardo G (2009). The Society of Thoracic Surgeons 2008 cardiac surgery risk models: part 2--isolated valve surgery. Ann Thorac Surg.

[3] Murkin JM, Adams SJ, Novick RJ (2007). Monitoring brain oxygen saturation during coronary bypass surgery: a randomized, prospective study. Anesth Analg.

[4] Murkin JM, Arango M (2009). Near-infrared spectroscopy as an index of brain and tissue oxygenation. Br J Anaesth.

[5] Ortega-Loubon C, Herrera-Gómez F, Bernuy-Guevara C (2019). Near-Infrared Spectroscopy Monitoring in Cardiac and Noncardiac Surgery: Pairwise and Network Meta-Analyses. J Clin Med.

[6] Yu Y, Zhang K, Zhang L (20181). Cerebral near-infrared spectroscopy (NIRS) for perioperative monitoring of brain oxygenation in children and adults. Cochrane Database of Systematic Reviews.

[7] Slater JP, Guarino T, Stack J (2009). Cerebral oxygen desaturation predicts cognitive decline and longer hospital stay after cardiac surgery. Ann Thorac Surg.

[8] Bennett SR, Smith N, Bennett MR (2020). Cerebral oximetry in adult cardiac surgery to reduce the incidence of neurological impairment and hospital length-of-stay: A prospective, randomized, controlled trial. J Intensive Care Soc.

[9] Chan MJ, Chung T, Glassford NJ, Bellomo R (2017). Near-Infrared Spectroscopy in Adult Cardiac Surgery Patients: A Systematic Review and Meta-Analysis. J Cardiothorac Vasc Anesth.

[10] Harhay MO, Ratcliffe SJ, Small DS, Suttner LH, Crowther MJ, Halpern SD (2019). Measuring and Analyzing Length of Stay in Critical Care Trials. Med Care.

[11] Shroyer LAW, Coombs LP, Peterson ED (2003). The Society of Thoracic Surgeons: 30-Day Operative Mortality and Morbidity Risk Models. Ann Thorac Surg.

[12] Heringlake M, Garbers C, Käbler JH (2011). Preoperative cerebral oxygen saturation and clinical outcomes in cardiac surgery. Anesthesiology.

[13] Moerman A, Vandenplas G, Bové T, Wouters PF, De Hert SG (2013). Relation between mixed venous oxygen saturation and cerebral oxygen saturation measured by absolute and relative near-infrared spectroscopy during off-pump coronary artery bypass grafting. Br J Anaesth.

